# Bellman’s GAP—a language and compiler for dynamic programming in sequence analysis

**DOI:** 10.1093/bioinformatics/btt022

**Published:** 2013-01-25

**Authors:** Georg Sauthoff, Mathias Möhl, Stefan Janssen, Robert Giegerich

**Affiliations:** ^1^Center of Biotechnology and Faculty of Technology, Bielefeld University, 33615 Bielefeld, Germany and ^2^Department of Computer Science and ^3^Centre for Biological Signalling Studies (BIOSS), Albert-Ludwigs-Universität 97110 Freiburg, Germany

## Abstract

**Motivation:** Dynamic programming is ubiquitous in bioinformatics. Developing and implementing non-trivial dynamic programming algorithms is often error prone and tedious. Bellman’s GAP is a new programming system, designed to ease the development of bioinformatics tools based on the dynamic programming technique.

**Results:** In Bellman’s GAP, dynamic programming algorithms are described in a declarative style by tree grammars, evaluation algebras and products formed thereof. This bypasses the design of explicit dynamic programming recurrences and yields programs that are free of subscript errors, modular and easy to modify. The declarative modules are compiled into C++ code that is competitive to carefully hand-crafted implementations.

This article introduces the Bellman’s GAP system and its language, GAP-L. It then demonstrates the ease of development and the degree of re-use by creating variants of two common bioinformatics algorithms. Finally, it evaluates Bellman’s GAP as an implementation platform of ‘real-world’ bioinformatics tools.

**Availability:** Bellman’s GAP is available under GPL license from http://bibiserv.cebitec.uni-bielefeld.de/bellmansgap. This Web site includes a repository of re-usable modules for RNA folding based on thermodynamics.

**Contact:** robert@techfak.uni-bielefeld.de

**Supplementary information:** Supplementary data are available at *Bioinformatics* online

## 1 MOTIVATION AND BACKGROUND

We review the difficulties experienced in the development of dynamic programming algorithms, and outline how Bellman’s GAP alleviates them.

### 1.1 Implementing dynamic programming algorithms

Bellman’s GAP is a programming system, which supports the development of dynamic programming (DP) algorithms over sequence data. In bioinformatics, such algorithms are ubiquitous, ranging from sequence alignments and RNA structure prediction to the predictions of RNA interactions or stochastic modeling based on hidden Markov models and stochastic context-free grammars (Durbin *et al.*, 1998).

The development of a DP algorithm traditionally consists of two stages. In the first, creative stage, the recursions of the algorithm are developed, and a scoring scheme is defined. In the second stage, the algorithm is implemented in an imperative programming language such as C, C++ or Java. This stage requires to intermingle recursions and scoring scheme for the sake of runtime efficiency, to decide about space-efficient allocation of tables for intermediate results, to dedicate a substantial amount of work to implement simple, full or even stochastic backtracing and may be to provide an efficient sliding-window version to search through large sequence data. All this requires algorithmic techniques that are known in principle, but must be recreated and adjusted with each application, and which are error-prone to implement and tedious to debug. Later modifications or extensions of the original design tend to affect many lines of code, creating new chances of error in already trusted code.

Bellman’s GAP supports both stages of DP algorithm development. It consists of the language GAP-L and the compiler GAP-C. GAP-L allows to compactly describe the design of DP algorithms in an abstract way. It enforces modularity of components, and thus fosters re-use and extension. GAP-C automatically generates a reasonably efficient C++ implementation of any algorithm specified in GAP-L, contributing the aforementioned implementation techniques.

Automating the implementation stage has a profound backward influence on the design stage. Models can be developed gradually, with their compiled implementations allowing us to test our ideas in many variants as they evolve. For each variant, alternative implementations can be tested by changing some compiler options. The recursions can, for example, either be evaluated bottom-up or top-down, the choice of DP tables can be varied, and both single thread and multi-threaded parallel code under the OpenMP standard can be generated where the scoring scheme allows for this.

### 1.2 Related work

Bellman’s GAP supports a specific programming method—DP over sequence data—but not a particular application domain. In this sense, it is similar to a compiler for linear algebra problems described in Fabregat-Traver and Bientinesi (2012), which reads equations and properties of the operators involved (such as symmetry, positive definiteness) and then transforms the equations to choose an efficient and numerically stable implementation from a large reservoir of numerical algorithms. This has applications, for example, in genome-wide association studies (GWAS), but it is not a GWAS tool. Similarly, Bellman’s GAP helps to implement flowgram denoising or RNA folding algorithms, but by itself, it is not a tool for next-generation read analysis or an RNA-specific tool. Domain-specific knowledge is brought in by the user. Depending how central the DP is in an application scenario, and depending on re-usable modules already available from earlier work in that domain, Bellman’s GAP may decrease overall work more or less.

In the recent Tornado software (Rivas *et al.*, 2012), a (context-free) grammar is at the heart of a declarative specification, whose implementation is automated.

Citing from Rivas *et al.* (2012), the TORNADO language is designed to provide compact grammar descriptions for a wide range of RNA structural features such as nearest-neighbor dependencies (e.g. stacking rules or mismatches), including higher-than-first-order dependencies, and parameterization of arbitrary loop length distributions.

In contrast, Bellman’s GAP seeks abstractness and modularity rather than compactness, and does not *per se* support a particular application domain. Input could be a biosequence as well as a sequence of matrix dimensions (matrix chain optimization), an arithmetic formula (El Mamun’s caravan) or a logical clause (3SAT-problem). Using explicit names for evaluation functions turns the grammar into a tree grammar, allowing to encapsulate domain-specific knowledge in evaluation algebras. For example, for RNA structure analysis, several grammars are available together with algebras for energy scoring, partition function, base pair counting, shape abstractions and structure output. To add in stochastic grammars, one needs to provide a stochastic evaluation algebra, together with support for parameter training. All algebras, as well as mechanisms for backtracing, sampling etc., can work with different grammars over the same signature, and owing to algebra products (see below), they can be used in combination.

Finally, let us remark that aside from Bellman’s GAP, two further implementation of the algebraic DP framework are currently under way in other groups.

### 1.3 Background: algebraic DP

We review, in an informal mode, the concepts of the *algebraic* discipline of DP (ADP), which circumscribe the scope of problems supported by Bellman’s GAP. See Giegerich *et al.* (2004) for the original definitions and Sauthoff *et al.* (2011) for their recent extensions, including the formal semantics of the GAP-L language.

DP algorithms evaluate a *search space* of solution *candidates*. A particular problem instance is given by an *input sequence*, or several thereof. A *scoring scheme* evaluates candidates in the search space and chooses from them the eventual solution(s), according to some *objective function*. Most often, this objective is score minimization or maximization, but counting candidates, enumeration and sampling from the search space are also common. Scoring scheme and objective function together constitute an *evaluation algebra*. The search space arising from concrete input is defined via a problem *decomposition* scheme, recursively splitting the input problem into smaller sub-problems. *Tabulation* is used to store and retrieve solutions from sub-problems, to avoid that they are evaluated multiple times. Finally, *Bellman’s principle of optimality* must hold. It ensures that the objective function distributes over scoring, and hence it suffices to consider only optimal solutions to sub-problems in search of the overall optimum. Other than this condition, there is no restriction on the objective functions that can be provided in an evaluation algebra.

In many applications, especially in biosequence analysis, the recursive problem decomposition naturally follows the sub-word structure of the input sequence(s). In this situation, any candidate can be described as a tree, composed of scoring functions, and bearing the input sequence at its leaves. *Evaluating* this tree, we get the candidate’s score.

**Fig. 1.**
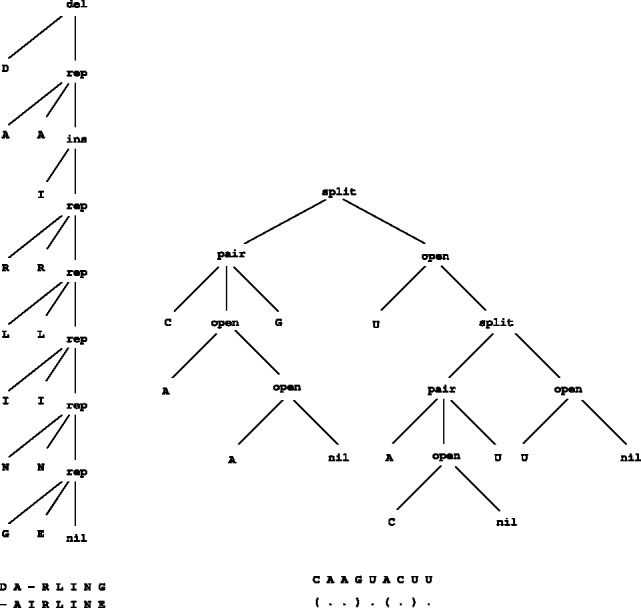
Left: tree representing an alignment of the amino acid sequences ‘DARLING’ and ‘AIRLINE’. *rep*(*lace*), *ins*(*ert*) and *del*(*ete*) denote the typical edit operations, and *nil* denotes an empty alignment. Right: tree representing a secondary structure assigned to an RNA sequence. *pair* indicates a base pair enclosing a sub-structure, *split* a branching structure, *open* an unpaired base next to a sub-structure, and *nil* the empty sub-structure

Figure 1 shows two such candidate trees, one representing an alignment of two short amino acid sequences and one representing a secondary structure of an RNA molecule. Substituting concrete functions for the tree symbols *rep*, *del*, *ins*, *nil*, say by the unit scoring scheme, the alignment candidate evaluates to a score of 3. Substituting base pair counting functions for *split*, *pair*, *open*, *nil*, the RNA structure candidate evaluates to its number of base pairs, which is 2 in our example.

Which candidate trees need to be considered for a given input? This is conveniently described by a tree grammar, which encodes the logic of problem decomposition in a natural top-down manner.

Sequence alignment by the Needleman–Wunsch algorithm (Needleman and Wunsch, 1970) is described by the grammar Alignment:


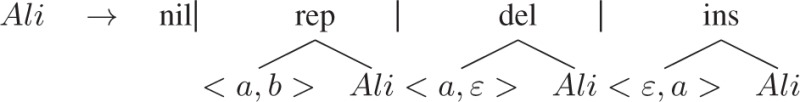


where *Ali* is the axiom of the grammar and the only non-terminal symbol, and *a*, *b* denote arbitrary characters from the underlying sequence alphabet.

RNA folding can be described by the grammar RNAStruct that reflects the algorithm of Nussinov *et al.* (1978):


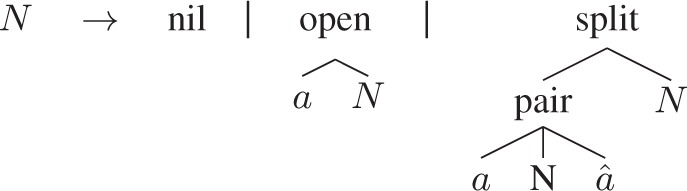


with the three cases for empty structures, structures where the first base *a* is unpaired and those where the first base *a* is paired with a complementary base 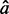.

Whatever our grammar is, for given input *x*, all the trees generated by the grammar, which spell out *x* at their leaves, constitute the search space. Grammar RNAStruct accepts a single input string, while grammar Alignment is a two-track grammar and expects two strings, indicated by the use of 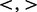.

A grammar and an evaluation algebra together precisely define the problem class to be solved. Expressed in GAP-L, all the technical ingredients of a DP algorithm—concrete recurrences, tabulation decisions, backtracing and more—can be generated automatically.

Having designed a tree grammar 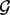 and an *evaluation algebra* 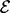, we simply compile. This turns the grammar into an evaluator of the search space defined by it, which by convention is also named 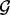. On input *x*, we call 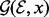 to obtain the desired solution.

Having conceptually separated candidate evaluation from search space construction allows us to create high-level operations on evaluation algebras, called products. These build new types of analysis from given components with a single keystroke, and no changes to tried-and-trusted code. Products as implemented in Bellman’s GAP are explained in Section 2.2. The name of Bellman’s GAP is derived from its key concepts: Bellman’s principle, Grammars, Algebras and Products.

### 1.4 Innovations in Bellman’s GAP

Bellman’s GAP is a third-generation system supporting algebraic DP. This discipline was originally implemented as a domain-specific language (Giegerich *et al.*, 2004), embedded in the lazy functional programming language Haskell. In the second implementation, ADP code was directly compiled into more efficient C code (Giegerich and Steffen, 2006), while the language itself was still a Haskell dialect. Its more widespread use was impeded by the relics of the Haskell embedding, such as a combinator syntax based on higher-order functions, which many potential users found obscure.

Bellman’s GAP brings about three major innovations compared with second-generation ADP:

- It completely breaks with its functional programming past by providing a C- or Java-style language, which nevertheless remains declarative.
- It extends the algebraic approach in significant ways (multi-track input, new algebra products).
- It generates faster and more space-efficient code than second-generation ADP.

## 2 THE BELLMAN’S GAP LANGUAGE AND COMPILER

### 2.1 GAP-L language features

The signature construct in GAP-L is similar to an interface declaration in Java. For the alignment example, we use

**signature** Align(**alphabet**, answer) {

 answer rep(<**alphabet**, **alphabet**>, answer);

 answer del(<**alphabet**, **void**>, answer);

 answer ins(<**void**, **alphabet**>, answer);

 answer nil(<**void**, **void**>);

 **choice** [answer] h([answer]);

}

With the name of the signature, generic names for the input alphabet and the results of candidate evaluation are specified. The declarations of the function symbols follow. The use of **void** indicates that del reads a character form the first track only, and ins only from the second; [and] indicates list types. The objective function, marked by **choice**, always computes on lists.

An algebra definition assigns concrete data types to the generic names and provides implementations for the function symbols. Algebra score uses distance minimization, and scores mismatches with 3 and gaps with 4:

**algebra** score **implements**

 Align(**alphabet** = **char**, answer = **int**) {

 **int** rep(<**char** a, **char** b>, **int** m)

 {**if** (a == b) **return** m; **else return** m + 3;}

 **int** del(<**char** g, **void**>, **int** m) {**return** m + 4;}

 **int** ins(<**void**, **char** g>, **int** m) {**return** m + 4;}

 **int** nil(<**void**, **void**>) {**return** 0;}

 **choice** [**int**] h([**int**] l) {**return** list(minimum(l));}

}

In the tree grammar, terminal symbols are, by convention, written in uppercase, and tree patterns are written like function applications:

**grammar** alignment **uses** Align(**axiom** = ali) {

 ali = rep(<CHAR, CHAR>, ali) |

 del(<gap, EMPTY>, ali) |

 ins(<EMPTY, gap>, ali) |

 nil(<EMPTY, EMPTY>) # h;

 gap = CHAR;

}

The operator 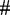 explicitly specifies that the objective function h is used to choose from alternative candidates.

Let print be another algebra that computes a visualization of a candidate as an alignment, i.e. the two input sequences padded with gaps. Used in a product such as

**instance** main = alignment(score * print);

we obtain the optimal score together with the candidates that achieve it. Each instance declaration implements a separate algorithm, for which GAP-C produces specialized code, depending on the algebras involved.

The signature for the RNA folding example is:

**signature** Nuss(**alphabet**, answer) {

 answer split(answer, answer);

 answer pair(**alphabet**, answer, **alphabet**);

 answer open(**alphabet**, answer);

 answer nil(**void**);

 **choice** [answer] h([answer]);

}

A simple algebra for base pair maximization is

**algebra** bpmax **implements**

 Nuss (**alphabet** = **char**, answer = **int**)

 **int** split (**int** x, **int** y) {**return** x+y;}

 **int** pair (**char** a, **int** x, **char** b) {**return** x+1;}

 **int** open (**char** a, **int** x) {**return** x;}

 **int** nil(**void**) {**return** 0;}

 **choice** [**int**] h ([**int** l]) {**return** list(maximum(l));

}

The RNAStruct grammar, which models the search space of all structures for the inut sequence, is a little bit more interesting than grammar alignment, as not all trees built from split, pair, open, nil are allowed:

**grammar** RNAStruct **uses** Nuss (**axiom**=N) {

 N = nil(EMPTY) |

 open(CHAR, N) |

 split(pair(CHAR, N, CHAR) **with** basep, N) # h;

}

This expresses that a candidate of the form

split(pair(CHAR, x, CHAR), pair(CHAR, y, CHAR))

is not welcome as part of the search space, as this structure already has a (unique) representation as

split(pair(CHAR, x, CHAR),

   split(pair(CHAR, y, CHAR), nil(EMPTY)))

In addition, the grammar further restricts the search space with the syntactic filter basep. This predicate guards the application of the tree pattern pair(CHAR, N, CHAR). It is only evaluated if the first and last characters are complementary to each other.

**algebra** cnt **auto** count

asks the compiler to generate an algebra cnt, which counts the number of candidates, and we leave it to the reader to write an algebra print that converts a candidate into a dot-bracket string (see also supplement). We declare two instances

**instance** cooptnum = RNAStruct(bpmax * cnt)

**instance** cooptimals = RNAStruct(bpmax * print)

where cooptnum determines the maximum number of base pairs, and the number of *different* structures that achieve it, and cooptimals computes and outputs all the co-optimal structures.

### 2.2 Products in GAP-L

Evaluation algebras have certain (non-exclusive) properties that are relevant when it comes to their use in combination. Algebra *A* is

- *unitary*, if it returns a single result (e.g. simple minimization, counting),
- *set valued*, if the computed multi-sets do not contain multiple entries (e.g. k-best minimization),
- *selective*, if the choice function always returns a sub-(multi-)set of a given multi-set (e.g. min(k) or enumeration),
- *enumerative*, if its choice function is 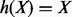,
- *synoptic*, if *h* computes new values from candidate values, which are not associated with a particular candidate (e.g. counting, summation, average).

These properties are used by the compiler for certain optimizations, but more importantly, they govern the use of products. Generally, a product in ADP is an operation that takes two algebras *A* and *B* over the same signature and creates combined algebra, say 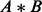. To achieve such an effect in traditional DP recurrences, almost every line of the code would have to be modified. Product operators allow to achieve this combination by a single keystroke.

Bellman’s GAP offers five product operators (see Table 1). Products can be combined with a user-defined semantic filter, using a suchthat clause. The use of these algebra products will be demonstrated in the examples below.

**Table 1.** Algebra products provided in Bellman’s GAP

Operator	Product name	Effect	Restrictions
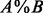	Cartesian	Cartesian product	*A*, *B* unitary
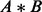	Lexicographic	Optimization under lexicographic ordering	*A*, *B* selective, *A* set valued
		classified DP	*A* enumerative
		reporting candidates	*B* enumerative
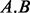	Take-one	Same as 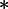 , suppressing co-optimals	*A* enumerative or set valued
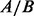	Interleaved	Optimization *across* classification	*A* enumerative or set valued
			*B* selective and non-empty
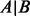	Overlay	Stochastic backtrace	*A* synoptic, *B* enumerative

See Section 4.2 for products in action.

### 2.3 Compiler features

The main task of the compiler GAP-C is to analyze the grammar and the algebra products used in instance declarations. From these, it derives recurrences (akin to a traditional formulation of a DP algorithm) for which imperative C++ code is generated.

As with the design of the language GAP-L, a goal of the compiler is to make ADP convenient to use. For this, the front-end of the compiler pedantically keeps track of source code locations to report parse errors to the column. In the middle-end, GAP-C includes semantic analyses for better diagnostics. For example, a type-checker pinpoints missing arguments or wrong types when using function symbols in algebras or the grammar. A product analysis checks whether a product is known to violate Bellman’s principle, and a runtime analysis phase computes the asymptotically optimal runtime and verifies whether the designer’s choice of table allocation (when explicitly given) actually achieves such a runtime.

We look at two of the optimizations that contribute to generating C++ target code that is competitive with handwritten one. For a deeper treatment, see Sauthoff *et al.* (2011).

GAP-C can generate code for different backtracing schemes. Backtracing is normally used to compute the structure of an optimal candidate (e.g. a string representation of an alignment or a secondary structure) after the optimal score is computed. Using backtracing is a performance optimization, as the candidate reconstruction is only done for those sub-solutions that are part of the optimal solutions. In the case of an alignment algorithm, this saves constant runtime factors and space; for RNA secondary structure prediction, the forward computation finding the maximum number of base pairs is in 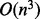, while the backtracing for the optimal candidate(s) is in 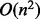.

For our first GAP-L code example, the product score * print computes the optimal alignment score and the alignment itself. With a compiler switch, GAP-C automatically generates backtracing code for the algebra print. Thus, only the algebra score is computed in the forward computation. By default, the backtracing code automatically outputs all co-optimal candidates. For the flowgram alignment variant below (Section 3.1), this is not necessary, and we can specify this via another compiler switch.

Further supported backtracing schemes are sub-optimal backtracing, backtracing over (*k*-best) classes during classification and stochastic backtracing (sampling) under an algebra that computes a probability distribution.

Another performance-critical optimization of GAP-C is table design. The table design phase automatically decides which intermediate results need to be stored in a table to achieve asymptotically optimal runtime, and which recurrences are not tabulated to save space. This problem is NP-hard in general. GAP-C uses a greedy heuristic that is safe (the asymptotically optimal runtime is always reached) and also takes certain constant runtime factors into account. Note that Bellman’s GAP cannot guarantee that your algorithm is efficient. If you specify a grammar with complexity 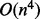 where an 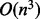 grammar is also possible, it is your fault. For a grammar as given, the code generated appears to be competent (see Section 4). Naturally, human dedication and tuning will eventually achieve performance superior to automatically generated code.

## 3 PROGRAM DEVELOPMENT IN GAP-L

In this section, we describe the implementation of a small, but ‘real-world’, application from scratch, and we document how software construction proceeds when we already have a repository of grammars and algebras. All the cited source files are contained in the supplement.

### 3.1 Flowgram denoising

Denoiser (Reeder and Knight, 2010) is a pipeline for reducing noise in reads from pyrosequencing samples. In pyrosequencing, a raw read is called a flowgram, which is a sequence of frames, where each frame consists of four floating point numbers. Each number codes the measured amount of light for the (non-)integration event of one of the four nucleotides during sequencing. Errors during pyrosequencing that lead, for example, to inserted or deleted frames in a flowgram are called noise. Taking flowgram data as-is in the analysis of meta-genomic samples would inflate the number of operational taxonomic units (Quince *et al.*, 2011). Denoiser clusters similar flowgrams together to reduce the noise.

As part of the clustering step, Denoiser computes sequence alignments of flowgrams. The authors used a custom Haskell embedding of algebraic DP to implement the alignment algorithm for rapid prototyping, easy evaluation of the scoring function and optimizations. The algorithm is sketched in Reeder and Knight (2010), but details are not given. In the following, we create a GAP-L version of the Reeder–Knight flowgram denoiser, using the basic pairwise alignment algorithm from Section 2.1 as starting point.

In the alignment of flowgrams, insertions or deletions refer to a whole frame, a sub-sequence of four numbers. We add two function symbols, ti for a terminal insertion and td for deletion, such that we can score them differently. The changes to the signature Align are:

**signature** FlowAlign(**alphabet**, answer) {

 answer rep(<**alphabet**, **alphabet**>, answer);

 answer del(<Subsequence, **void**>, answer);

 answer ins(<**void**, Subsequence>, answer);

 answer nil(<**void**, **void**>);

 answer ti(<**void**, **int**>);

 answer td(<**int**, **void**>);

 **choice** [answer] h([answer]);

}

After that, we have to adjust the grammar to make use of the modified and added function symbols. In case of an insertion or deletion, the grammar has to parse exactly four numbers of a sequence. We model this with syntactic filters that restrict the size of the parsed sub-sequence:

**grammar** flow **uses** FlowAlign(**axiom** = ali) {

 gap = REGION **with** minsize(4) **with** maxsize(4);

 ali = {rep(<CHAR, CHAR>, ali) |

 del(<gap, EMPTY>, ali) |

 ins(<EMPTY, gap>, ali) |

 nil(<EMPTY, EMPTY>) |

 ti(< EMPTY, SEQ >) |

 td(< SEQ, EMPTY >)

 } **with** banded(10, 20, 20) # h;

}

With terminal insertions or deletions, we only need their length (and not their concrete sequence); thus, we can use the terminal parser SEQ, which returns the length of the parsed sub-sequence.

In Denoiser, the rep case allows for frame shifting, in contrast to indels that are restricted to the frame size. To modify this decision from Reeder and Knight (2010), we can easily adjust the grammar and the scoring algebra.

For performance reasons, Denoiser provides a banded version of the alignment algorithm. Banded DP means that only the entries in the score matrix around the diagonal in a band of width *k* are computed. This is appropriate for aligning flowgrams because scores of dissimilar flowgrams are not needed in the clustering. In GAP-L, we can derive a banded version of the algorithm with the syntactic filter banded, which guards the application of the alternatives of the non-terminal ali. The syntactic filter banded here considers only entries that are in the band 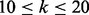 with 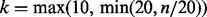, where *n* denotes the flowgram length.

The other existing rules of the grammar do not need to be changed because the terminal parser CHAR is independent of the used alphabet, i.e. with the alphabet of floating point numbers, the CHAR terminal parser returns a valid floating pointer from the input.

In Denoiser, the scoring scheme for flowgram alignment uses four components. We optimize with one scheme and compute two relative scores. We model such scoring with four algebras: score, length, mismatch and seqlen. The scoring algebra score does minimization and looks up mismatch scores in a table.

**algebra** score **implements**

 FlowAlign(**alphabet** = **float**, answer = **float**) {

 **float** rep(<**float** a, **float** b>, **float** m)

 {**return** m + score_table(a, b);}

 **float** del(<Subsequence g, **void**>, **float** m)

 {**return** m + 15.0 * 4.0;}

 **float** ins(<**void**, Subsequence g>, **float** m)

 {**return** m + 15.0 * 4.0;}

 **float** nil(<**void**, **void**> {**return** 0.0;}

 **float** ti(<**void**, **int** b>) {**return** 0.0;}

 **float** td(<**int** a, **void**>) {**return** 0.0;}

 **choice** [**float**] h([**float**] l){**return** list(minimum(l));}

}

The length algebra length computes the length of the alignment without counting a terminal insertion or deletion. We leave the definition of the algebra as an ‘exercise’ to the reader (the algebra is included in the supplement). Thus, we can use the result of the product

score * length

to compute a relative flowgram alignment score. However, the optimization is based on algebra score alone, and algebra length computes additional information for each optimal candidate.

The mismatch algebra directly scores distances between (mis)matching characters (i.e. numbers) and sums up the values of inserted or deleted characters:

**algebra** mismatch **implements**

 FlowAlign(**alphabet** = **float**,

 answer = **int**) {

 **int** rep(<**float** a, **float** b>, **int** m)

 {**return** m + abs(round(a) - round(b));}

 **int** del(<Subsequence g, **void**>, **int** m)

 {**return** m + round(g[0]) + round(g[1])

 + round(g[2]) + round(g[3]);}

 **int** ins(**<void**, Subsequence g>, **int** m)

 {**return** m + round(g[0]) + round(g[1])

 + round(g[2]) + round(g[3]);}

}

 **choice** [**int**] h([**int**] l)

 {**return** list(minimum(l));}

For computing a relative mismatch score, we use the algebra seqlen that scores like mismatch except for the replace case. In GAP-L, we can directly communicate this via modifying the existing algebra:

**algebra** seqlen **extends** mismatch {

 **int** rep(<**float** a, **float** b>, **int** m)

 {**return** m + max(round(a), round(b));}

}

In Denoiser, the flowgram alignment component provides a mode to compute both relative scores simultaneously, which can be achieved with the following product:

**instance** RKdenoiser =

 flow(score * (length % mismatch % seqlen))

This specifies that for the candidate with the minimal score, also the alignment length, the mismatch score and mismatch length are computed. In all four algebras, insertions and deletions at the terminal end are scored zero, as flowgrams may differ in size, but only an insertion or deletion at the 5′ end is plausible with this input data. In this way, we compute a variant of a local alignment algorithm.

### 3.2 Programming with modular components

Working in a specific application area, one builds up a library of modules—signatures, grammars and algebras—from which a variety of tasks can be compiled using algebra product. Each novel algebra product gives rise to a program instance (cf. Section 2.1), from which the compiler produces an executable specialized to this task.

Our repository for RNA folding is a result of the study in Janssen *et al.* (2011). It holds separate collections of signatures, grammars and algebras, and GAP-L main programs that merely consist of instance declarations. Among others, the repository contains the modules shown in Table 2. Counting algebras are not in the repository, as they are constructed by the compiler on request. Instances of the form

1. nodangle (shape_i*pfunc **suchthat** pfunc_filter)

for 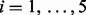 were used in Janssen *et al.* (2011) for all four grammars to evaluate the effect of different treatments of dangling bases on the shape probabilities. The **suchthat** clause activates a filter that weeds out shapes with accumulated probabilities <10^−^^6^, which was shown to speed up the computation without significantly distorting results. Instance

(2) nodangle(((shape5*(mfe %pfunc)) **suchthat** pfunc_filter_allPP)*dotBracket)

must be compiled with option –kbacktrace, adds in the computation of mfe structures for each shape and reports them via algebra dotBracket. This is an interesting use of the Cartesian product, where two independent results for each shape are computed simultaneously, the shape’s mfe value and the accumulated contribution of all structures with this shape to the partition function. Instance

(3) nodangle(((pfunc|pfunc_id)*(shape2* mfe*dotBracket)) **suchthat** sample_filter)

is compiled with option –sample. It computes a stochastic sample from the partition function, where for each candidate sampled, its level 2 shape, free energy and dot-bracket string are reported. This allows to estimate shape probabilities by sampling and comparing them with their exact computation via (1).

(4) Finally, the general technique of *classified* DP is realized as follows. Consider an algebra *A*, which computes from each candidate a classification attribute, not performing any optimization. This splits the candidate space into disjoint classes. Then, a product of form (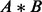) performs classified DP, where the analysis carried out by *B* (which can be a simple algebra as well as an arbitrary product) applies to each class separately.

**Table 2.** Modules related to RNA structure prediction based on thermodynamics

Type	Name	Purpose
Signature	foldrna	Describes RNA folding space
Grammar	nodangle	Model without dangling bases
Grammar	overdangle	Model with overaggressive dangling
Grammar	microstate	Correct dangling with…
Grammar	macrostate	…Without candidate space extension
Grammar	nodangle_lp	Model allowing lonely pairs
Algebra	mfe	MFE computation
Algebras	pfunc	Boltzmann weights (partition function)
	pfunc_id	Individual candidate Boltzmann weight
Algebras	shapes1…5	Classification by shape abstraction
Algebra	dotBracket	Printing structures

From the viewpoint of software technology, the important aspect here is that each algebra is designed and tested with a single purpose in mind. Then, product operations allow to define more sophisticated analyses in great variety—without making changes to existing modules.

## 4 EVALUATION OF BELLMAN’S GAP AS AN IMPLEMENTATION PLATFORM

In this section, we evaluate Bellman’s GAP based on our experience of using it in bioinformatics tool development.

### 4.1 Code quality

**Fig. 2.**
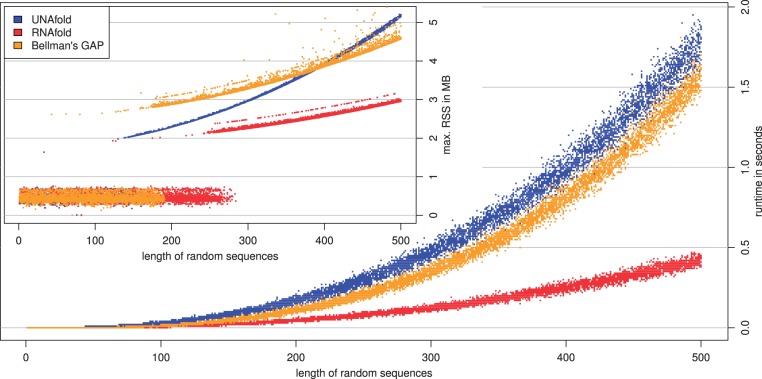
Measurement on 10 000 uniformly distributed random sequences with length between 1 and 500 bases. See text for discussion. Runtime (user + system) and memory consumption (max, RSS) measured by UNIX tool ‘memtime’ (by Johan Bengtsson). RNAfold version 2.0.2 -d 1 –noLP –noPS, UNAfold: hybrid-ss-min –suffix = DAT –mfold –NA = RNA –tmin = 37 –tinc = 1 –tmax = 37 –sodium = 1 –magnesium = 0 -I, Bellman’s GAP: mfe * dotBracket –backtrace -t microstate grammar. Short sequence memory consumption seems to fit into initial stack/heap size of an OS process

Our goal is not to reproduce existing tools with more efficient code, but to ease development of novel applications. Still, the generated code must reach efficiency comparable with hand-coded implementations. In terms of asymptotics, the compiler always achieves the best possible asymptotic runtime (cf. Sauthoff *et al.*, 2011), but also makes considerations about space consumption and constant factors. We compare against the hand-crafted applications UNAfold and RNAfold. Note that UNAfold is rather new, whereas RNAfold has been carefully engineered over more than a decade and has the reputation of being the fastest RNA folding program. For comparison, we use instance microstate(mfe * dotbracket) for MFE structure prediction. This is equivalent with RNAfold, but also reports co-optimals. Measurements show that in terms of speed, Bellman’s GAP code lies between the hand-coded tools (Fig. 2). Memory consumption is similar to UNAfold, with a tendency to be more space efficient on longer sequences. Note the outlier points above the yellow memory curve. These can be traced back to sequences that have several different co-optimal structures. Especially with grammar microstate, their number can be large; the maximum observed in this data set was 384 for a sequence of length 476. Our automatic backtrace generates a somewhat wasteful list of all co-optimals in such a case before producing output.

Bellman’s GAP has been extensively evaluated against the second-generation implementation of ADP. The tool RNAshapes predicts RNA secondary structure classified by abstract shapes (Steffen *et al.*, 2006), and the tool pknotsRG predicts structures including simple pseudoknots and kissing hairpin interactions (Theis *et al.*, 2010). Both were originally implemented in the Haskell-embedded version of ADP. They were re-coded in GAP-L and compiled with GAP-C, achieving constant-factor speed-ups. pknotsRG, for example, is two to three times as fast while using about half the space. For more details, see Sauthoff (2011).

### 4.2 Assessing convenience

In the previous sections, we have described how abstractness, modularity and automated compilation help to achieve reliable implementations with less human effort. This cannot be quantified easily. As a crude approximation to implementation effort, we here compare code sizes for ‘real-world’ applications.

In Janssen *et al.* (2011), the impact of four different implementations of the Turner energy model of RNA folding was investigated. These implementations differ for pragmatic reasons with respect to their treatment of dangling bases. RNAfold, from the Vienna package (Lorenz *et al.*, 2011), provides three variants via options -d0, -d1 and -d2, called *NoDangle*, *Microstate* and *OverDangle* for comparison, respectively, while RNAshapes uses a fourth model, *Macrostate*. We compared models by measuring their effect on shape probabilities, which provides a more robust measure than single minimum free energy structure prediction. Because RNAfold does not compute shape probabilities, rather than augmenting the source code from the Vienna package, it was easier for us to re-implement all three versions in Bellman’s GAP, and combine them with probabilistic shape analysis by a product of algebras already available from RNAshapes.

Table 3 reports on the size of the grammars and algebras arising for these applications. We specify sizes of grammar and algebras, as well as the size of the generated code for certain instances. Note the significant difference between the number of grammar non-terminals (recurrences) and the number of tables actually allocated to compute them. The code size reduction (size of GAP-L code/size of C++ code) gives an idea of how much programming and debugging time is saved in ‘real-world’ scenarios by using Bellman’s GAP. In the table, we included, from our ongoing research, a remake of the Rfam model RF00553. Covariance models are specialized toward a particular structure, leading to grammars with hundreds of non-terminal symbols and rules. The example is included to show that Bellman’s GAP successfully handles grammars of this size.

**Table 3.** GAP-L program sizes and target code sizes

Tool	Problem solved	No. of algebra functions	No. of algebras used	No. of NTs in 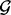	No. of distinct cases	No. of NTs tabulated	Lines of code GAP-L	Lines of code C++
RNAshapes	Shape representative structures of RNA	19	3	11	29	5	487	5456
RNAshapes	Probabilistic shape analysis	36	2	26	72	15	640	4796
pknotsRG	Pseudoknot prediction	38	2	25	63	19	755	9581
GAP-RNAfold	RNA folding (RNAfold -d0 emulation)	15	2	11	25	5	191	3719
RF00553	Covariance model remake	258	2	80	257	77	3348	51 646

The number of cases distinguished in the problem decomposition is defined as the number of right-hand sides over all rules of the tree grammar. Lines of code are given for a typical instance using a product of two or three algebras, such as 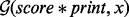.

## 5 CONCLUSION

### 5.1 Adopting Bellman’s GAP in education

At Bielefeld and Freiburg universities, starting 2011, DP based on Bellman’s GAP has been taught to small cohorts of bioinformatics M.Sc. students. This paragraph reports from our teachers’ observations; it does not pretend to be a formal evaluation of student success. Our students have a limited experience in DP the ‘hard’ way, and generally find it rewarding to invest in the intellectual overhead of adopting the algebraic discipline of DP. Bellman’s GAP as a language appears to be a minor problem, owing to its small size and resemblance of C and Java. A didactic advantage is the high-level error messages generated by the compiler. They lead to less frustration and debugging time among students and reduce supervision required by the teacher. Aside from type errors and missing arguments, the compiler detects also infinite cyclic recursions and warns when some recursion leads to exponential complexity. In early assignments, we rely on automatic table design feature by GAP-C, allowing to keep things simple at the beginning. Later, when space efficiency concerns are covered in the lecture, students design their own table allocation and compare it with the one automatically derived by the compiler. Abstract specifications and ease of implementation also foster a deeper understanding and encourage experimentation. How is the size of the search space affected by splitting a sub-case in two separate cases? A counting algebra provides the answer. Are the shape representative structures the same under two different scoring schemes? Calling nodangle(shape * (score1 % score2). dotbracket) generates first evidence. In this manner, exploration of modeling alternatives is supported.

### 5.2 Scope of applications

We circumscribe the scope of Bellman’s GAP by some present and forthcoming applications.

- The present article uses problems in sequence alignment, flowgram denoising and thermodynamic RNA folding for the exposition. Illustrating the use of algebra products, several problem variants were addressed herein.
- The core algorithms of RNAshapes have been re-coded and run in Bellman’s GAP, and were used for the study by Janssen *et al.* (2011). (The full user interface is still lacking.)
- These RNA folding modules can also be used to create thermodynamic matchers for structural RNA motifs by writing motif-specific grammars, as recently reported from an RNA-seq study in *Sinorhizobium meliloti* (Reinkensmeier *et al.*, 2011).
- While this manuscript was under review, two novel applications of Bellman’s GAP have appeared, one on RNA structure analysis (Huang *et al.*, 2012) and one on minisatellite map alignment (Löwes, 2012). The latter provides a non-ambiguous version of the algorithm by Abouelhoda *et al.* (2009), and is technically interesting because it combines a two-track problem (minisatellite aligment) with a non-trivial single-track sub-problem (reconstruction of duplication histories).
- In our ongoing work on covariance models, we extend their concept to support more than one—similar, but not identical—consensus structure(s). Stochastic parameters, trained with a two-track grammar, are used in a Viterbi-like scoring algebra, akin to pfunc_id (cf. Table 2). Inside scoring is simply achieved by replacing choice function max with sum, akin to pfunc.
- In ongoing work, we are adapting the Locomotif tool by Reeder *et al.* (2007) to the MicroState grammar of Janssen *et al.* (2011) to generate motif matchers coded in GAP-L from user-supplied structure graphics. Future users of Locomotif will be customers of Bellman’s GAP, possibly without noticing it.

Bellman’s GAP is strictly limited to problems over (one or more) sequences. While it seems possible to extend the fundamental concepts of ADP to tree-structured inputs, such as tree edit distance or tree alignment algorithms, no automated implementation for such a more powerful framework is presently within reach.

### 5.3 Future development as a community effort

In contrast to the majority of tools in bioinformatics, which serve biologists to analyze their data, Bellman’s GAP is a tool for bioinformaticians. We have reported our experience, by which Bellman’s GAP helps to create more reliable and more versatile DP algorithm in a shorter time. By the first release of the Bellman’s GAP system, we hope to share this experience with the bioinformatics community, and together create libraries of re-usable specifications for a variety of applications. To this end, we have created a repository of Bellman’s GAP modules related to thermodynamic folding and abstract shape analysis of RNA. The repository named fold-grammars as well as the Bellman’s GAP system are available open source under GPL license from http://bibiserv.cebitec.uni-bielefeld.de/bellmansgap.

## Supplementary Material

Supplementary Data

## References

[btt022-B1] Abouelhoda MI (2009). Alignment of minisatellite maps based on run-length encoding scheme. J. Bioinform. Comput. Biol..

[btt022-B2] Durbin R (1998). Biological Sequence Analysis.

[btt022-B3] Fabregat-Traver D, Bientinesi P (2012). A domain-specific compiler for linear algebra operations. High Performance Computing for Computational Science—VECPAR 2012.

[btt022-B4] Giegerich R, Steffen P, Haddad H (2006). Challenges in the compilation of a domain specific language for dynamic programming. Proceedings of the 2006 ACM Symposium on Applied Computing.

[btt022-B5] Giegerich R (2004). A discipline of dynamic programming over sequence data. Sci. Comput. Program..

[btt022-B6] Huang J (2012). Abstract folding space analysis based on helices. RNA.

[btt022-B7] Janssen S (2011). Lost in folding space? Comparing four variants of the thermodynamic model for RNA secondary structure prediction. BMC Bioinformatics.

[btt022-B8] Lorenz R (2011). ViennaRNA package 2.0. Algorithms for Mol. Biol..

[btt022-B9] Löwes B (2012). Analysis of minisatellite sequences with Algebraic Dynamic Programming in Bellman’s GAP.

[btt022-B10] Needleman SB, Wunsch CD (1970). A general method applicable to the search for similarities in the amino acid sequence of two proteins. J. Mol. Biol..

[btt022-B11] Nussinov R (1978). Algorithms for loop matchings. SIAM J. Appl. Math..

[btt022-B12] Quince C (2011). Removing noise from pyrosequenced amplicons. BMC Bioinformatics.

[btt022-B13] Reeder J, Knight R (2010). Rapidly denoising pyrosequencing amplicon reads by exploiting rank-abundance distributions. Nat. Methods.

[btt022-B14] Reeder J (2007). Locomotif: from graphical motif description to RNA motif search. Bioinformatics.

[btt022-B15] Reinkensmeier J (2011). Conservation and occurrence of trans-encoded srnas in the rhizobiales. Genes.

[btt022-B16] Rivas E (2012). A range of complex probabilistic models for RNA secondary structure prediction that includes the nearest-neighbor model and more. RNA.

[btt022-B17] Sauthoff G (2011). Bellman’s GAP: A 2nd Generation Language and System for Algebraic Dynamic Programming.

[btt022-B18] Sauthoff G (2011). Bellman’s GAP: a declarative language for dynamic programming. Proceedings of the 13th International ACM SIGPLAN Symposium on Principles and Practices of Declarative Programming.

[btt022-B19] Steffen P (2006). RNAshapes: an integrated RNA analysis package based on abstract shapes. Bioinformatics.

[btt022-B20] Theis C (2010). Prediction of RNA secondary structure including kissing hairpin motifs. Proceedings of the 10th Workshop on Algorithms in Bioinformatics (WABI 2010) LNBI 6293.

