# Supplementary material for: Bellman’s GAP—a language and compiler for dynamic programming in sequence analysis
Source: Bioinformatics. 2013 Jan 25;29(5):551–60. doi: 10.1093/bioinformatics/btt022 (PMC3582264; doi:10.1093/bioinformatics/btt022)
Supplement: Supplementary Data [file supp_29_5_551__index.html]

Bellman’s GAP—a language and compiler for dynamic programming in sequence analysis — Bellman’s GAP—a language and compiler for dynamic programming in sequence analysis — Supplementary Data 

# Bellman’s GAP—a language and compiler for dynamic programming in sequence analysis

## Supplementary Data

files

**Files in this Data Supplement:**

- Supplementary Data - gz file
