# Supplementary material for: Bellman’s GAP—a language and compiler for dynamic programming in sequence analysis
Source: Bioinformatics. 2013 Jan 25;29(5):551–60. doi: 10.1093/bioinformatics/btt022 (PMC3582264; doi:10.1093/bioinformatics/btt022)
Supplement: Supplementary Data [file supp_btt022_bgsuppl.tar.gz › bgsuppl/suppl_interact.pdf]

SUPPLEMENT

This suplement holds

1. another educational example of GAP-L code for a simple RNA-RNA interaction problem
2. the complete source code of the four programming examples presented in the body of the paper, and in this supplement.

All four examples are listings of the GAP-L source files, ready to compile. They are included to demonstrate that with Bellman's GAP, there is no programming effort other than providing the declarative components of a GAP-L program: signatures, algebras, grammars, and instances.

Readers may also use this code as a starting point for their own experiments with alternative scoring models, more refined search spaces, more advanced flowgram denoisers, and so on.

RNA interaction example

To demonstrate a two-tape RNA problem, we give a toy example for RNA interaction We extend the RNASTRUCT grammar to compute the joint structure of two RNAs whose external loops may interact. Since interacting RNA strands run in opposite direction, one of the two inputs must be given in 3' – 5' order. See Figure 1 for a simple example. We extend the grammar with the following rules for the additional nonterminals N' and I, where I is now the axiom.

$I \rightarrow$ nil' | interact($<a, \hat{a}>$, I) | (1)

strucL($<N', \epsilon>$, I) | strucR($<\epsilon, N'>$, I) $\ldots h$

$N' \rightarrow$ open(a, N) | split(pair(a, N, $\hat{a}$), N) $\ldots h$ (2)

$N \rightarrow$ ... as before (3)

N' is identical to N except that it does not include the empty structure. The nonterminal I (for Interaction) relies on the feature of GAP-L to support multiple input tracks. Therefore, it has leaves of the form $<t_1, t_2>$, where t_1 and t_2 are terminals or nonterminals representing a part of the first and second track, respectively.

The four cases in the decomposition for I are

- the base case of two empty sequences/structures,
- a structure where the first base a of x interacts with the first (and complementary) base $\hat{a}$ of y,
- a structure where the beginning of the first sequence has some structure that does not include interactions.
- a structure where the beginning of the second sequence has some structure that does not include interactions.

Since GAP-L allows to mix multi-track and single-track rules, the cases for I can use N' to represent arbitrary structures on a single track. Note that this simple grammar does only allow for non-crossing, external loop interactions.

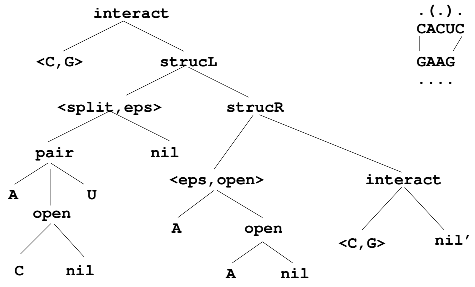

Fig. 1. Two interacting RNAs (upper right corner) and their corresponding tree.

Assuming that we want to give interaction base pairs only a score of 0.8 instead of 1 we extend the bpmax as follows.

$$\text{nil'} = 0 \qquad \text{interact}(<a, \hat{a}>, x) = x + 0.8$$
$$\text{strucL}(<x, \epsilon>, y) = x + y \qquad \text{strucR}(<\epsilon, x>, y) = x + y$$

All existing algebra functions of bpmax can be re-used for the interaction variant without any modifications.

The concrete GAP-L file for this example is included in this supplement with thw other source files.

RNA Interaction

```
/* RNA Interaction


This is an educational example demonstrating a two
track folding problem.
The grammar is most simple and not suitable for
engergy-based scoring. Note also that the grammar is
ambiguous, cf. note below.
*/


import interact
        // auxiliary definitions in C++
        // (e.g. filter basepair, reading bases
        // from DIFFERENT tracks)

input < raw, raw >
        // no special encoding, two track input


type spair = ( string first, string second )
        // data type for pretty print result

signature Interact(alphabet, answer) {

  answer nil(void);
  answer unpaired(answer, alphabet);
  answer pair(alphabet, answer, alphabet);
  answer split(answer, answer);

  answer nil2(<void,void>);
  answer interact(answer,<alphabet,alphabet>);
  answer struct1(answer,<answer,void>);
  answer struct2(answer,<void,answer>);

  choice [answer] h([answer]);

}

algebra bpmax
        // simple base pair maximization
  implements Interact(alphabet = char, answer = int)
{
  int nil(void)
  {
    return 0;
  }

  int unpaired(int a, char c)
  {
    return a;
  }

  int pair(char c, int m, char d)
  {
    return m + 1;
  }

  int split(int l, int r)
  {
    return l + r;
  }

  int nil2(<void, void>)
  {
      return 0;
  }

  int interact(int a, <char c, char d>)
        // interacting base pairs are here given the
        // same weight as intramolecular base pairs
  {
      return a + 1;
  }

  int struct1(int a, <int b, void>)
  {
      return a + b;
  }

  int struct2(int a, <void, int b>)
  {
      return a + b;
  }

  choice [int] h([int] l)
  {
    return list(maximum(l));
  }

}

algebra dotbracket
        // creates two dotbracket strings
  implements Interact(alphabet = char, answer = spair ) {
  spair nil(void)
  {
      spair r;
      return r;
  }

  // single sequences always use first component of pair
  spair unpaired(spair a, char c)
  {
      spair r;
      append(r.first,a.first);
      append(r.first,'.');
      return r;

  }

  spair pair(char c, spair m, char d)
  {
    spair r;
    append(r.first,'(');
    append(r.first,m.first);
    append(r.first,')');
    return r;
  }

  spair split(spair a, spair b)
  {
    spair r;
    append(r.first,a.first);
    append(r.first,b.first);
    return r;
```

```
  }

  spair nil2(<void, void>)
  {
    spair r;
    return r;
  }

  spair interact(spair a, <char c, char d>)
        // interactions in the external loop
        // are printed as '|' in both structures
  {
    spair r;
    append(r.first,a.first);
    append(r.second,a.second);
    append(r.first,'|');
    append(r.second,'|');
    return r;
  }

  spair struct1(spair a, <spair b, void>)
  {
    spair r;
    append(r.first,a.first);
    append(r.second,a.second);
    append(r.first,b.first);
    return r;
  }

  spair struct2(spair a, <void, spair b>)
  {
    spair r;
    append(r.first,a.first);
    append(r.second,a.second);
    append(r.second,b.first);
    return r;
  }

  choice [spair] h([spair] l)
  {
    return l;
  }
}

algebra enum  auto enum;
algebra count auto count;
        // automatically generated algebras

grammar interaction uses Interact (axiom=I) {
        // derived from single seq. grammar
        // S -> nil | S a | S a S a'

  I = nil2(<EMPTY,EMPTY>) |
      interact(I,<CHAR,CHAR> with basepair ) |
        // interaction only in external loop
      struct1(I,<Nnonempty,EMPTY>) |
      struct2(I,<EMPTY,Nnonempty>) # h;
        // these two rules are the source
        // of a grammar ambiguity --
        // check it out using instance enum!

  N = nil(EMPTY) | Nnonempty # h;
        // a intramolecular substructure must
        // be non-empty,

  Nnonempty = unpaired(N, CHAR) |
              split(N, bp) # h ;

  bp = pair(CHAR, N, CHAR) with char_basepairing ;

}

instance bpmax   = interaction( bpmax );
instance print   = interaction( dotbracket );
instance bpmaxpp = interaction( bpmax * dotbracket );
instance enum    = interaction( enum );
```

Sequence Alignment

```
// Sequence Alignment (see paper text)

input < raw, raw >
        // no special encoding, two track input

type spair = ( string first, string second )

signature Align(alphabet, answer) {
  answer rep( <alphabet, alphabet>, answer );
  answer del( <alphabet, void>, answer);
  answer ins( <void, alphabet>, answer);
  answer nil( <void, void>);
  choice [answer] h([answer]);
}

algebra score implements
        // simple distance scoring
    Align(alphabet = char, answer = int) {
  int rep( <char a, char b>, int m)
  {
    if (a == b)
      return m;
    else
      return m + 3;
  }

  int del( <char g, void>, int m)
  {
    return m + 4;
  }

  int ins( <void, char g>, int m)
  {
    return m + 4;
  }

  int nil( <void, void> )
  {
    return 0;
  }

  choice [int] h([int] l)
  {
    return list(minimum(l));
  }
}

algebra print
        // produces a string pair, i.e.

        // the two lines of the alignment
  implements Align(alphabet = char, answer = spair )
{
  spair rep(< char a, char b>, spair m)
  {
    spair r;
    append(r.first, a);
    append(r.first, m.first);
    append(r.second, b);
    append(r.second, m.second);
    return r;
  }

  spair del(<char a, void>,  spair m)
  {
    spair r;
    append(r.first, a);
    append(r.first, m.first);
    append(r.second, '-');
    append(r.second, m.second);
    return r;
  }

  spair ins(<void , char b>, spair m)
  {
    spair r;
    append(r.first, '-');
    append(r.first, m.first);
    append(r.second, b);
    append(r.second, m.second);
    return r;
  }

  spair nil(<void, void>)
  {
    spair r;
    return r;
  }

  choice [spair] h([spair] l)
  {
    return l;
  }

}


grammar alignment uses Align(axiom = ali)
        //simple edit model with singleton gaps
{
  ali = rep( <CHAR, CHAR>, ali)   |
        del( <gap, EMPTY>, ali)   |
        ins( <EMPTY, gap>, ali)   |
        nil( <EMPTY, EMPTY> )     # h ;
  gap = CHAR ;
}

instance score   = alignment(score) ;
instance scorepp = alignment(score * print) ;
```

RNA Structure

```
// RNA Structure (see paper text)

signature Nuss(alphabet, answer) {
        // the four structural features of
        // the simplest possible model for RNA structure
  answer split(answer, answer);
  answer pair(alphabet, answer, alphabet);
  answer open(alphabet, answer);
  answer nil(void);
  choice [answer] h([answer]);

}

algebra print
        // produces a dot-bracket string
  implements Nuss(alphabet = char, answer = string)
{

  string split(string l, string r)
  {
    string res;
    append(res, l);
    append(res, r);
    return res;
  }

  string pair(char c, string m, char d)
  {
    string r;
    append(r, '(');
    append(r, m);
    append(r, ')');
    return r;
  }

  string open(char c, string a)
  {
    string r;
    append(r, '.');
    append(r, a);
    return r;
  }

  string nil(void)
  {
    string r;
    return r;
  }

  choice [string] h([string] l)
  {
    return l;
  }

}

algebra bpmax
        // counts base pairs and maximizes
  implements Nuss(alphabet = char, answer = int)
{
  int split(int x, int y)
  {
    return x + y;
  }

  int pair(char a, int x, char b)
  {
    return x + 1;
  }

  int open(char a, int x)
  {
    return x;
  }

  int nil(void)
  {
    return 0;
  }


  choice [int] h([int] l)
  {
    return list(maximum(l));
  }

}

algebra count auto count;
        // counts candidiates
algebra enum auto enum;
        // outputs candidates as trees

        // While the count algebra can be generated,
        // here comes its hand-coded equivalent

algebra mycount
         // counts candidates in the search space
  implements Nuss(alphabet = char, answer = int)
{
  int split(int x, int y)
  {
    return x * y;
  }

  int pair(char a, int x, char b)
  {
    return x;
  }

  int open(char a, int x)
  {
    return x;
  }

  int nil(void)
  {
    return 1;
  }


  choice [int] h([int] l)
  {
    return list(sum(l));
  }
}
```

```
algebra mycount_id extends mycount
        // to be used with sampling structures
        // with even probability
{
  choice [int] h([int] l)
  {
    return l;
  }
}



grammar RNAStruct uses Nuss (axiom=N) {
        // a non-ambiguous version of the
        // classical Nussinov recurrence
  N = nil(EMPTY) |
          open(CHAR, N) |
          split(pair(CHAR, N, CHAR)
               with char_basepairing,N) # h ;
}

instance print = RNAStruct( print ) ;

instance bpmax = RNAStruct( bpmax ) ;

instance bpmaxpp = RNAStruct( bpmax * print ) ;

instance count = RNAStruct(count);

instance bpmaxcnt = RNAStruct( bpmax * count ) ;

instance sampleEqual =
    RNAStruct(((mycount | mycount_id) * print)
        suchthat sample_filter);
```

Flowgram Alignment

```
// Flowgram Alignment (see paper text)

import helper
        // auxiliaries coded in C++ (myround, banded)

input < raw, raw >
        // no special encoding, two track input

type Rope = extern
        // predefined type for string operations
type spair = ( Rope first, Rope second )

signature FlowAlign(alphabet, answer) {
        // classical alignment model ...
  answer rep( <alphabet, alphabet>, answer );
  answer del( <Subsequence, void>, answer);
  answer ins( <void, Subsequence>, answer);
  answer nil( <void, void>);

  answer ti( <void, int>);
        // extended by terminal insertions ...
  answer td( <int, void>);
        // ... and deletions
  choice [answer] h([answer]);
}

algebra count auto count ;
        // produce counting algebra
algebra enum auto enum;
        // produce (tree) enumeration algebra

algebra print
        // produces a pair of strings, i.e.
        // the two lines of the alignment
  implements FlowAlign(alphabet = float,
                       answer = spair ) {

  spair rep( <float a, float b>, spair m)
  {
    spair r;
    append(r.first, a);
    append(r.first, ' ');
    append(r.first, m.first);

    append(r.second, b);
    append(r.second, ' ');
    append(r.second, m.second);
    return r;
  }

  spair del( <Subsequence g, void>, spair m)
  {
    spair r;
    append(r.first, '*', size(g));
    append(r.first, m.first);
    append(r.second, "- - - - ");
    append(r.second, m.second);
    return r;
  }

  spair ins( <void, Subsequence g>, spair m)
  {
```

```
    spair r;
    append(r.first, "- - - - ");
    append(r.first, m.first);
    append(r.second, '*', size(g));
    append(r.second, m.second);
    return r;
  }

  spair nil( <void, void> )
  {
    spair r;
    return r;
  }

  spair ti( <void, int s> )
        // terminal insertions/deletion are
        // shown as "="
  {
    spair r;
    append(r.second, '=', s);
    return r;
  }

  spair td( <int s, void> )
  {
    spair r;
    append(r.first, '=', s);
    return r;
  }


  choice [spair] h([spair] l)
  {
    return l;
  }

}

algebra score
        // basic scoring algebra, here
        // the only algebra used for optimization
  implements FlowAlign(alphabet = float,
                       answer = float ) {

  float rep( <float a, float b>, float m)
  {
    return m + score_table(a, b);
  }

  float del( <Subsequence g, void>, float m)
  {
    return m + 15.0 * 4.0;
  }

  float ins( <void, Subsequence g>, float m)
  {
    return m + 15.0 * 4.0;
  }

  float nil( <void, void> )
  {
    return 0.0;
  }

  float ti( <void, int b>)
  {
    return 0.0;
  }

  float td( <int a, void>)
  {
    return 0.0;
  }

  choice [float] h([float] l)
  {
    return list(minimum(l));
  }

}
        // The following algebras compute
        // additional info about the candidate,
        // they are not used during optimization

algebra length_alg
        // computes alignment length
  implements FlowAlign(alphabet = float,
                       answer = int ) {

  int rep(<float a, float b>, int m)
  {
    return m + 1;
  }

  int del(<Subsequence g, void>, int m)
  {
    return m + 4;
  }

  int ins(<void, Subsequence g>, int m)
  {
    return m + 4;
  }

  int nil(<void, void>)
  {
    return 0;
  }

  int ti(<void, int b>)
  {
    return 0;
  }

  int td(<int a, void>)
  {
    return 0;
  }

  choice [int] h([int] l)
  {
    return list(minimum(l));
  }

}

algebra mismatch
        // computes mismatch score
```

```
  implements FlowAlign(alphabet = float,
                       answer = int ) {

  int rep(<float a, float b>, int m)
  {
    return m + abs(myround(a) - myround(b));
  }

  int del(<Subsequence g, void>, int m)
  {
    return m
      + myround(g[0])+ myround(g[1])
      + myround(g[2]) + myround(g[3]);
  }

  int ins(<void, Subsequence g>, int m)
  {
    return m
      + myround(g[0]) + myround(g[1])
      + myround(g[2]) + myround(g[3]);
  }

  int nil(<void, void>)
  {
    return 0;
  }

  int ti(<void, int b>)
  {
    return 0;
  }

  int td(<int a, void>)
  {
    return 0;
  }

  choice [int] h([int] l)
  {
    //return l;
    return list(minimum(l));
  }

}
```

```
algebra seqlen extends mismatch {
        // accumulatesa a positive weight
        // for matched residues
  int rep(<float a, float b>, int m)
  {
    return m + max(myround(a), myround(b));
  }
}


grammar flow uses FlowAlign(axiom = ali)
        // alignment grammar
{

  gap = REGION with minsize(4) with maxsize(4) ;
        // gaps retain reading frame

  ali =  {
        rep( <CHAR, CHAR>, ali)     |
        del( <gap, EMPTY>, ali)    |
        ins( <EMPTY, gap>, ali)    |
        nil( <EMPTY, EMPTY> )      |

        ti( < EMPTY, SEQ > )       |
        // insert/delete overhang
        td( < SEQ, EMPTY > )
          } with banded(10, 20, 20)
        // user defined syntactic filter
                                  # h ;

}

instance score = flow(score);
instance print = flow(print);
instance sp = flow(score*print);


instance count = flow(count);
instance enum = flow(enum);

instance fga =
  flow(score * (length_alg % mismatch % seqlen));
```
